# Decitabine-Intensified Modified Busulfan/Cyclophosphamide Conditioning Regimen Improves Survival in Acute Myeloid Leukemia Patients Undergoing Related Donor Hematopoietic Stem Cell Transplantation: A Propensity Score Matched Analysis

**DOI:** 10.3389/fonc.2022.844937

**Published:** 2022-03-16

**Authors:** Ziying Li, Wei Shi, Xuan Lu, Hui Lu, Xiena Cao, Liang Tang, Han Yan, Zhaodong Zhong, Yong You, Linghui Xia, Yu Hu, Huafang Wang

**Affiliations:** Institute of Hematology, Union Hospital, Tongji Medical College, Huazhong University of Science and Technology, Wuhan, China

**Keywords:** decitabine, busulfan, cyclophosphamide, acute myeloid leukemia, related donor hematopoietic stem cell transplantation

## Abstract

To identify the benefit of decitabine (Dec)-intensified myeloablative conditioning on the outcomes of patients with acute myeloid leukemia (AML) after related donor hematopoietic stem cell transplantation (HSCT), we performed a retrospective matched‐pair study from a pool of 156 patients to evaluate Dec [20 mg/m^2^/day intravenously (i.v.) on days −11 to −7]-intensified modified busulfan/cyclophosphamide (mBuCy) conditioning regimen vs. mBuCy regimen in 92 AML patients, with 46 patients in each cohort. The cumulative incidence of grade II–IV acute graft-versus-host disease (aGVHD) was lower in the Dec group (15.2% ± 0.3% vs. 32.6% ± 0.5%, *P* = 0.033). Compared with mBuCy group (15.5% ± 0.3%), a significantly higher proportion of limited chronic GVHD (cGVHD) in Dec group (35% ± 0.6%) was observed (*P* = 0.025). Dec-intensified mBuCy conditioning was associated with better 2-year overall survival (OS) and GVHD-free relapse-free survival (GRFS) (81% ± 6.2% vs. 59.4% ± 7.5%, *P* = 0.03; 58.7% ± 8.1% vs. 40.9% ± 7.3%, *P* = 0.042; respectively). Our results also elucidated that the Dec group had better 2-year OS and lower 2-year cumulative incidence of relapse (CIR) in patients acquiring haploidentical HSCT than that of the mBuCy group (84.8% ± 7.1% vs. 58.2% ± 10.3%, *P* = 0.047; 17.9% ± 0.8% vs. 40.0% ± 1.0%, *P* = 0.036; respectively), which did not increase the treatment-related mortality and regimen-associated toxicities. Dec-intensified myeloablative regimen and high-risk stratification were the variables associated with OS, leukemia-free survival (LFS), and GRFS in multivariate analysis. In high-risk patients, no differences were found in CIR, OS, LFS, and GRFS between the two groups. These data indicated that Dec-intensified mBuCy conditioning regimen was associated with better survival than mBuCy regimen in AML patients, especially in patients undergoing haploidentical HSCT.

## Introduction

Allogeneic hematopoietic stem cell transplantation (allo-HSCT) is the backbone curative treatment for acute myeloid leukemia (AML) patients, especially for high-risk patients (1). Due to rapid hemopoietic rehabilitation and low transplant-related mortality (TRM), modified busulfan/cyclophosphamide (mBuCy) regimen has become more common than BuCy for myeloablative conditioning (MAC) in AML patients undergoing HSCT in China (2–6).

Recently, intensive MAC such as intensification of BuCy with fludarabine (7), cladribine (8), idarubicin (9), or decitabine (Dec) (10) has been explored.

Dec (5-aza deoxycytidine) acts as an inhibitor of DNA methyltransferase I, resulting in the reexpression of silent antioncogenes and the terminal differentiation of leukemic cells (11). Furthermore, Dec can mitigate graft-versus-host disease (GVHD) through enhancing the effect of T regulatory lymphocytes (12). For patients with myeloid malignancies, some clinical studies have demonstrated that Dec-modified conditioning regimens were associated with lower relapse rate (RR), reduced incidence and severity of GVHD, and satisfactory survival in allo-HSCT settings (13–18).

A previous study observed durable response rate for high-dose Dec-intensified BuCy regimen in high-risk AML patients undergoing human leukocyte antigen (HLA)-identical sibling donor (ISD) transplantation (19). For high-risk AML patients not in remission, low-dose Dec in combination with mBuCy was associated with lower RR and better survival (10). Thus, Dec booster BuCy regimen has become a more effective conditioning regimen in patients with high-risk AML. However, the value of Dec-intensified regimen has not been compared with those of mBuCy for intermediate- and high-risk AML patients who underwent related donor HSCT.

Herein, we retrospectively performed a matched‐pair study to clarify the association of Dec-intensified mBuCy conditioning regimen with the outcomes of intermediate- and high-risk AML patients undergoing either an ISD or a haploidentical donor (HID) transplantation.

## Methods

### Patients

A cohort of 156 patients who were diagnosed with *de novo* AML undergoing related donor HSCT at our center between March 2012 and November 2020 was enrolled in this study. Among them, 58 patients had received Dec in combination with mBuCy as the conditioning regimen (Dec group), and 98 who received the mBuCy regimen were selected as the control group (mBuCy group). High-risk AML included the following: hyperleukocytosis at diagnosis, no response to induction chemotherapy, ≥complete response 2 (CR2) or not in remission, extramedullary leukemia, relapse within 6 months from induction or consolidation therapy, ≥two relapses or relapse after auto-HSCT, and high-risk cytogenetics according to the National Comprehensive Cancer Network 2018 guidelines (www.nccn.org) (20, 21). Patients who had ≥5% BM blasts in pretransplantation were classified as having active disease (NR).

All patients or their legal guardians provided signed informed consent for the use of protected health data for research, as approved by the Institutional Review Board on Medical Ethics at Tongji Medical College of Huazhong University of Science and Technology and which has been conducted in accordance with the Declaration of Helsinki.

### Conditioning Regimen

The mBuCy regimen was as follows: oral hydroxycarbamide 80 mg/kg/day orally on day −9, cytarabine 2 g/m^2^ intravenously (i.v.) every 12 h on day −8, busulfan 3.2 mg/kg/day i.v. on days −7 to −5, cyclophosphamide 1.8 g/m^2^/day i.v. on days −4 and −3, and semustine (Me-CCNU) 250 mg/m^2^/day orally on day −2. Dec-intensified mBuCy regimen included Dec 20 mg/m^2^/day i.v. on days −11 to −7, cytarabine 1.5 g/m^2^ i.v. every 12 h on days −9 to −7, followed by busulfan 3.2 mg/kg/day i.v. on days −6 to −4 and cyclophosphamide 1.8 g/m^2^/day i.v. on days −3 and −2, and Me-CCNU 250 mg/m^2^/day orally on day −1. Antithymocyte globulin (Thymoglobulin; Sanofi Aventis, Paris, France) was used as part of conditioning in HID-HSCT as previously reported (22). Supportive care was administered as described previously (21).

### Stem Cell Mobilization and Collection

Donor bone marrow (BM) stem cells and/or peripheral blood stem cells (PBSCs) were collected using standard mobilization protocols. All donors were given recombinant human granulocyte colony-stimulating factor (rhG-CSF) 8–10 μg/kg/day to mobilize BM and/or peripheral blood. For HLA-identical sibling and HLA 4–5/6 or 6–8/10 matched related donor transplantation, unmanipulated rhG-CSF-mobilized PBSCs were infused into the recipient on the day of collection. After removal of plasma and some of the red blood cells, the BM collections were resuspended in human albumin and infused.

### Graft-Versus-Host Disease Prophylaxis

Cyclosporin A (CsA) with short-term methotrexate (MTX) was given to patients who received matched related donor transplantation. A regimen of tacrolimus (FK506), short-term MTX, mycophenolate mofetil (MMF), and basiliximab was given to patients who received haploidentical transplantation. Dosage adjustments of CsA or FK506 were based on plasma target levels of 150–250 or 7–15 ng/ml, respectively. CsA or FK506 was tapered and discontinued after 3–4 months in cases without GVHD. MTX was administered i.v. at dosages of 15 mg/m^2^ on day +1 and 10 mg/m^2^ on days +3, +6, and +11. MMF (7.5 mg/kg, twice daily) was given orally from day −9 and tapered and discontinued after 2 months. Basiliximab was given i.v. at a dose of 20 mg on day 0 (2 h before graft infusion) and day +4.

### Relapse Monitor, Prophylaxis, and Preemptive

All patients had BM examinations and minimal residual disease (MRD) evaluation by quantitative real-time PCR (qRT-PCR) or/and multiparametric flow cytometry (MFC) performed at 1, 2, 3, 4, 5, 6, 9, and 12 months after allo-HSCT and at 6-month intervals thereafter. Prevention of posttransplant hematologic or cytogenetic relapse includes immunosuppressant withdrawal and multiple consolidation chemotherapy and therapeutic donor lymphocyte infusion, with basic reference to Huang’s protocol (23).

### Endpoints

The evaluation of hematopoietic reconstitution was similar to that reported previously (24). Neutrophil engraftment was defined as an absolute neutrophil count >0.5 × 10^9^/L for 3 consecutive days posttransplantation. Platelet engraftment was defined as an absolute platelet count >20 × 10^9^/L without platelet transfusion for 7 consecutive days. Regimen-related toxicities were graded by common criteria according to Bearman et al. (25). The primary endpoints of the study were overall survival (OS), leukemia-free survival (LFS), and cumulative incidence of relapse (CIR). Secondary endpoints were TRM, GVHD-free relapse-free survival (GRFS), and cumulative incidence of GVHD. OS was defined as the time from transplant to death or last follow-up. LFS was defined as survival without evidence of relapse or progression. Relapse was based on standard hematologic criteria. TRM was defined as death in complete remission (CR). GRFS was defined as being alive without relapse and without grade III–IV acute GVHD (aGVHD) and/or chronic GVHD (cGVHD) requiring immunosuppressive treatment (26). The clinical diagnosis of aGVHD was performed according to consensus criteria (27). cGVHD was defined using published criteria (28).

### Propensity Score Matching and Statistical Analysis

We used propensity score matching (PSM) (29, 30) to obtain bias-corrected comparisons of the transplant outcomes. The following matching variables were used as covariates: age and sex of patients, risk stratification, disease status before transplantation, disease course before transplantation, donor/recipient sex match, donor HLA type, source of stem cells. A 1:1 nearest neighbor matching by propensity score was performed within a caliper of 0.2 standard deviations. A propensity score test (**Figure S1**) confirmed the normalizing effect of covariate matching.

Descriptive statistics are presented as median values and ranges for continuous variables and percentages for categorical variables. Comparisons of variables between the two groups were performed using chi-square test, Fisher’s exact test, Student’s t-test, or Mann–Whitney U-test. LFS, OS, and GRFS were calculated by the Kaplan–Meier method and compared using the log-rank test. The cumulative incidence of TRM, relapse, and GVHD was estimated using the Gray’s test considering competing risks. TRM and relapse were the competing risks. For aGVHD and cGVHD, the competing events were relapse and death. The Cox proportional hazards model was used for multivariate analysis. All *P* < 0.1 in CIR, OS, LFS, or GRFS univariate analysis was defined for selecting variables for entry into the multivariate model. All *P* values were two-sided, and *P* < 0.05 was considered significant. Statistical analyses were performed with SPSS 24.0 (SPSS Inc./IBM, Armonk, NY, USA), R 4.1.2 (R Development Core Team, Vienna, Austria), and the R package MatchIt.

## Results

### Patient Characteristics

All patient characteristics are summarized in **Table S1**. Among the overall cohort, the variables of patient age, disease status, prior lines of therapy, disease course, donor age, donor/recipient sex match, ABO match, stem cell source, and median follow-up time were comparable between the Dec group and mBuCy group, but the characteristics of sex (*P* = 0.014), risk stratification (*P* < 0.001), and donor HLA type (*P* = 0.028) were significantly different.

To overcome the imbalance among the aforementioned baseline variables, PSM was introduced and 46 case-control pairs (92 patients) were selected. After PSM, potential selection bias related to the two groups was minimized (**Table 1**; all *P* > 0.05).

**Table 1:** Characteristics of matched patients’ cohort.

Variable	Dec (n = 46)	mBuCy (n = 46)	*P* value
Median age, years (range)	32 (14–56)	40 (15–61)	0.224
Sex (n, %)			0.129
Female	13 (28.3)	7 (15.2)	
Male	33 (71.7)	39 (84.8)	
Risk stratification (n, %)			0.677
Intermediate-risk	22 (47.8)	24 (52.2)	
High-risk	24 (52.2)	22 (47.8)	
Disease status before HSCT (n, %)			1.000
CR_MRD-_	40 (87.0)	41(89.1)	
CR_MRD+_	3 (6.5)	3 (6.5)	
NR	3 (6.5)	2 (4.4)	
Median number of prior lines of therapy (range)	3 (1–8)	3 (1–8)	0.714
Disease course before HSCT (n, %)			1.000
≤12 months	42 (91.3)	42 (91.3)	
>12 months	4 (8.7)	4 (8.7)	
Donor age, years, median (range)	41 (9–58)	33 (10–57)	0.197
Donor/recipient sex match (n, %)			1.000
Female-male	13 (28.3)	13 (28.3)	
No Female-male	33 (71.7)	33 (71.7)	
ABO match (n, %)			0.954
Matched	24 (52.2)	25 (54.4)	
Major mismatched	11 (23.9)	12 (26.1)	
Minor mismatched	8 (17.4)	7 (15.2)	
Bidirect mismatched	3 (6.5)	2 (4.3)	
Donor HLA type (n, %)			0.527
HLA-identical sibling	18 (39.1)	21 (45.7)	
HLA-antigen mismatched related	28 (60.9)	25 (54.3)	
Stem cell source (n, %)			0.778
PBSC	39 (84.8)	38 (82.6)	
PBSC+BM	7 (15.2)	8 (17.4)	
Median follow-up time, months, (range)	24 (1–85)	23 (2–106)	0.601

Dec, decitabine; mBuCy, modified busulfan/cyclophosphamide; HSCT, hematopoietic stem cell transplantation; CR, complete remission; MRD, minimal residual disease; NR, active disease; PBSC, peripheral blood stem cell; BM, bone marrow.

### Engraftment

The neutrophil engraftment in Dec and mBuCy groups occurred at 12 days (range, 9–22) and 11 days (range, 8–22), respectively. The corresponding engraftment rates were 95.7% and 100% (*P* = 0.475). Platelet engraftment time was 13 days (range 5–21) in the Dec group and 11 days (range 7–26) in the mBuCy group, with engraftment rates of 93.5% and 97.8% (*P* = 0.609), respectively. In the Dec group, graft failure was observed in 1 patient, and this patient died 34 days after allo-HSCT.

### Early Regimen-Related Toxicities

Most cases experienced mild and moderate regimen-related toxicities. Toxicities involving the gastrointestinal tract were most common, including mild nausea/vomiting (Dec group, 45.7%; mBuCy group, 50.0%; *P* = 0.676) and diarrhea (Dec group, 21.7%; mBuCy group, 34.8%; *P* = 0.165). Oropharyngeal mucositis was more often observed in Dec-intensified conditioning regimen. Twenty-nine cases (63.0%) of oropharyngeal mucositis (58.7% grade I–II and 4.3% grade III) were observed in Dec group vs. 16 cases (34.8%) in mBuCy group (32.6% grade I–II and 2.2% grade III) (*P* = 0.007). Reversible elevations of liver enzymes were also seen in the two groups (Dec group, 10.9%; mBuCy group, 4.3%; *P* = 0.432). One patient in the Dec group had a seizure that was relieved by early intervention. Regimen-related cardiovascular or renal complications were not observed.

### Infectious Complications

The incidences of cytomegalovirus (CMV) antigenemia, hemorrhagic cystitis, and respiratory infection for the Dec vs. mBuCy group were 43.5% vs. 60.9% (*P* = 0.095), 15.2% vs. 13.0% (*P* = 0.765), and 23.9% vs. 19.6% (*P* = 0.613), respectively (**Table 2**). One patient in the Dec group developed CMV-associated interstitial pneumonia.

**Table 2:** Incidence of transplantation-related infectious complications.

	Dec	mBuCy	*P* value
CMV viremia	20 (43.5%)	28 (60.9%)	0.095
Hemorrhagic cystitis	7 (15.2%)	6 (13.0%)	0.765
Respiratory infection	11 (23.9%)	9 (19.6%)	0.613

Dec, decitabine; mBuCy, modified busulfan/cyclophosphamide; CMV, cytomegalovirus.

### Graft-Versus-Host Disease

**Figure 1:**
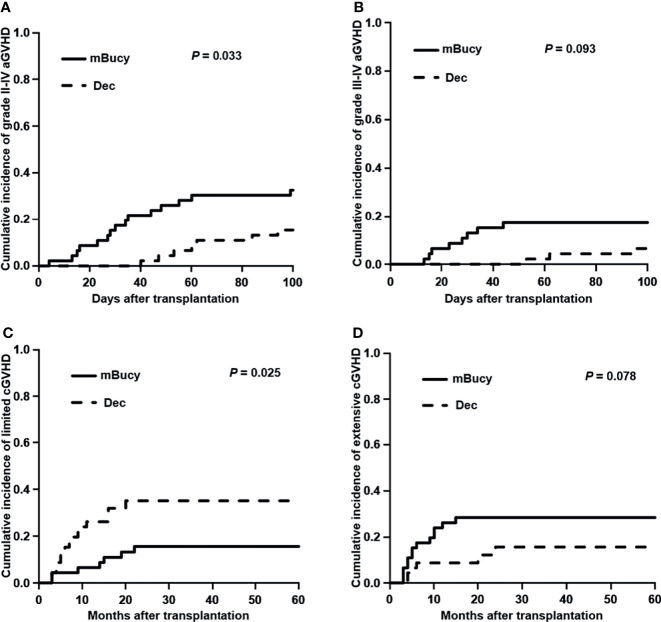
Cumulative incidence of **(A)** grade II–IV acute graft-versus-host disease (aGVHD), **(B)** grade III–IV aGVHD, **(C)** limited chronic GVHD (cGVHD), and **(D)** extensive cGVHD in the decitabine (Dec) group and the modified busulfan/cyclophosphamide (mBuCy) group.

The 100-day cumulative incidence of grade II–IV aGVHD was 15.2% ± 0.3% in the Dec group and 32.6% ± 0.5% in the mBuCy group (*P* = 0.033; **Figure 1A**). However, the 100-day cumulative incidence of grade III–IV aGVHD was not statistically different between Dec and mBuCy groups: 6.5% ± 0.1% vs. 17.4% ± 0.3% (*P* = 0.093; **Figure 1B**).

The 2‐year cumulative incidence of limited cGVHD was 35.0% ± 0.6% in the Dec group and 15.5% ± 0.3% in the mBuCy group (*P* = 0.025; **Figure 1C**). The 2‐year cumulative incidence of extensive cGVHD was identical between Dec and mBuCy groups: 15.6% ± 0.4% vs. 28.4% ± 0.5% (*P* = 0.078; **Figure 1D**).

### Relapse Incidence and Transplant-Related Mortality

**Figure 2:**
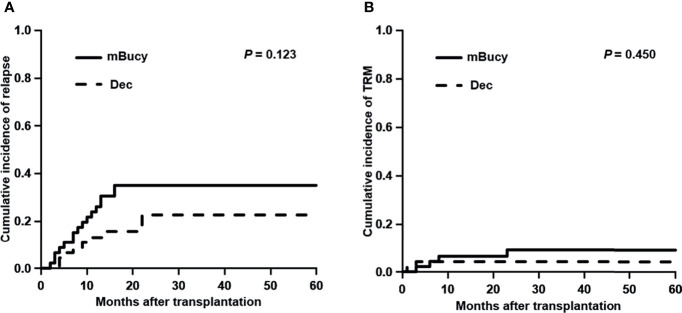
Cumulative incidence of **(A)** relapse and **(B)** transplant-related mortality (TRM) in the decitabine (Dec) group and the modified busulfan/cyclophosphamide (mBuCy) group.

At the time of last follow-up, 27 patients were in relapse, including 10 in the Dec group and 17 in the mBuCy group, and median time intervals from transplantation to relapse were 10 months (range, 4–25) and 9 months (range, 2–39) (*P* = 0.815). No difference in the 2-year CIR (22.6% ± 0.5% vs. 35.1% ± 0.5%; *P* = 0.123) was observed (**Figure 2A**). Causes of death are shown in **Table 3**. The 2‐year cumulative incidence of TRM was 4.3% ± 0.1% in the Dec group and 9.2% ± 0.2% in the mBuCy group (*P* = 0.450; **Figure 2B**).

**Table 3:** Primary causes of death in the study cohort.

	Dec	mBuCy
No. of patients	10	21
Relapse (n, %)	8 (80.0%)	16 (76.2%)
TRM (n, %)		
aGVHD	0	0
cGVHD	0	2 (9.5%)
Rejection	1 (10.0%)	0
Infection	1 (10.0%)	2 (9.5%)
Other	0	1 (4.8%)

Dec, decitabine; mBuCy, modified busulfan/cyclophosphamide; TRM, treatment-related mortality; aGVHD, acute graft-versus-host disease; cGVHD, chronic graft versus host disease.

### Survival

**Figure 3:**
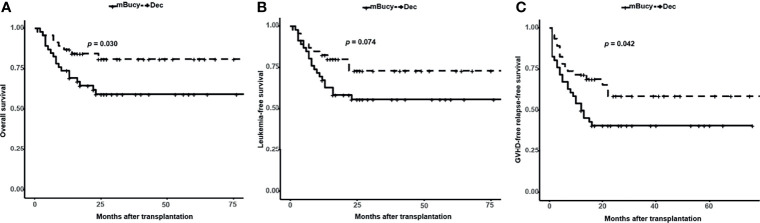
Probability of **(A)** overall survival, **(B)** leukemia-free survival, and **(C)** graft-versus-host disease (GVHD)-free relapse-free survival in the decitabine (Dec) group and the modified busulfan/cyclophosphamide (mBuCy) group.

The median follow-up in the Dec group was 24 months (range, 1–85), whereas for the mBuCy group was 23 months (range, 2–106). Two-year OS and GRFS were significantly improved in the Dec group than those in the mBuCy group (2-year OS, 81.0% ± 6.2% vs. 59.4% ± 7.5%, *P* = 0.030; 2-year GRFS, 58.7% ± 8.1% vs. 40.9% ± 7.3%, *P* = 0.042; **Figures 3A, C** **)**. Two-year LFS of the Dec group was similar to that of mBuCy group (73.1% ± 7.2% vs. 55.8% ± 7.4%, *P* = 0.074; **Figure 3B**).

### Univariate Analysis and Multivariate Analysis

Univariate analysis (**Table 4**) showed that risk stratification was important for all outcomes (CIR, *P* = 0.009; OS, *P* = 0.019; LFS, *P* = 0.010; GRFS, *P* = 0.012). Disease status before HSCT may be more susceptible to affect OS (*P* = 0.043). The conditioning regimen containing Dec had a beneficial influence on OS (*P* = 0.031), LFS (*P* = 0.016), and GRFS (*P* = 0.043). Additionally, donor/recipient sex match was associated with CIR (*P* = 0.029).

**Table 4:** Univariate analysis for relapse, OS, LFS, and GRFS.

Variable	To CIR	To OS	To LFS	To GRFS
	*P* value	*P* value	*P* value	*P* value
Age (≤40 years/>40 years)	0.996	0.889	0.683	0.993
Sex (male/female)	0.697	0.679	0.742	0.163
Risk stratification (intermediate/high)	0.009	0.019	0.01	0.012
Disease status before HSCT (CR_MRD-_/CR_MRD+_/NR)	0.478	0.043	0.106	0.132
Disease course before HSCT (≤12 months/>12 months)	0.452	0.36	0.343	0.876
Donor/recipient sex match (female-male/others)	0.029	0.186	0.103	0.291
Donor HLA type(HLA-identical sibling/HLA-antigen mismatched related)	0.698	0.522	0.527	0.209
Stem cell source (PBSC/PBSC+BM)	0.819	0.731	0.923	0.612
Conditioning regimen (Dec/mBuCy)	0.13	0.031	0.016	0.043
aGVHD grades II–IV	0.237	0.921	0.889	
aGVHD grades III–IV	0.75	0.748	0.802	
cGVHD	0.924	0.634	0.686	
Limited cGVHD	0.866	0.375	0.415	
Extensive cGVHD	0.766	0.142	0.174	

CIR, cumulative incidence of relapse; OS, overall survival; LFS, leukemia-free survival; GRFS, graft-versus-host disease-free relapse-free survival; HSCT, hematopoietic stem cell transplantation; CR, complete remission; MRD, minimal residual disease; NR, active disease; PBSC, peripheral blood stem cell; BM, bone marrow; Dec, decitabine; mBuCy, modified busulfan/cyclophosphamide; aGVHD, acute graft-versus-host disease; cGVHD, chronic graft-versus-host disease.

These results prompted us to conduct a multivariate analysis (**Table 5**), which confirmed that high-risk stratification was associated with a higher CIR (HR = 3.124; 95% CI: 1.338–7.293; *P* = 0.008), a lower OS (HR = 0.386; 95% CI: 0.177–0.842; *P* = 0.017), a lower LFS (HR = 0.398; 95% CI: 0.189–0.839; *P* =0.015), and a lower GRFS (HR = 0.500; 95% CI: 0.267–0.933; *P* = 0.029). The conditioning regimen containing Dec had a positive impact on OS (HR = 2.402; 95% CI: 1.123–5.137; *P* = 0.024), LFS (HR = 2.047; 95% CI: 1.009–4.154; *P* = 0.047), and GRFS (HR = 1.836; 95% CI: 1.011–3.334; *P* = 0.046). Female donor/male recipient was associated with a lower CIR (HR = 0.258; 95% CI: 0.077–0.861; *P* = 0.028). However, disease status did not affect OS (HR = 1.392; 95% CI: 0.465–4.162; *P* = 0.554).

**Table 5:** Multivariate analysis for relapse, OS, LFS, and GRFS.

Variable	CIR		OS		LFS		GRFS	
	HR (95% CI)	*P*	HR (95% CI)	*P*	HR (95% CI)	*P*	HR (95% CI)	*P*
Risk stratification	3.124 (1.338–7.293)	0.008	0.386 (0.177–0.842)	0.017	0.398 (0.189–0.839)	0.015	0.500 (0.267–0.933)	0.029
Disease status before HSCT	1.550 (0.452–5.309)	0.485	1.392 (0.465–4.162)	0.554	1.025 (0.382–2.750)	0.961	0.728 (0.309–1.716)	0.468
Donor/recipient sex match	0.258 (0.077–0.861)	0.028	0.496 (0.202–1.218)	0.126	0.455 (0.187–1.108)	0.083	0.721 (0.352–1.479)	0.372
Conditioning regimen	0.513 (0.234–1.125)	0.096	2.402 (1.123–5.137)	0.024	2.047 (1.009–4.154)	0.047	1.836 (1.011–3.334)	0.046

CIR, cumulative incidence of relapse; OS, overall survival; LFS, leukemia-free survival; GRFS, graft-versus-host disease-free relapse-free survival; HR, hazard ratio; CI, confidence interval; HSCT, hematopoietic stem cell transplantation.

### Subgroup Analysis

**Figure 4:**
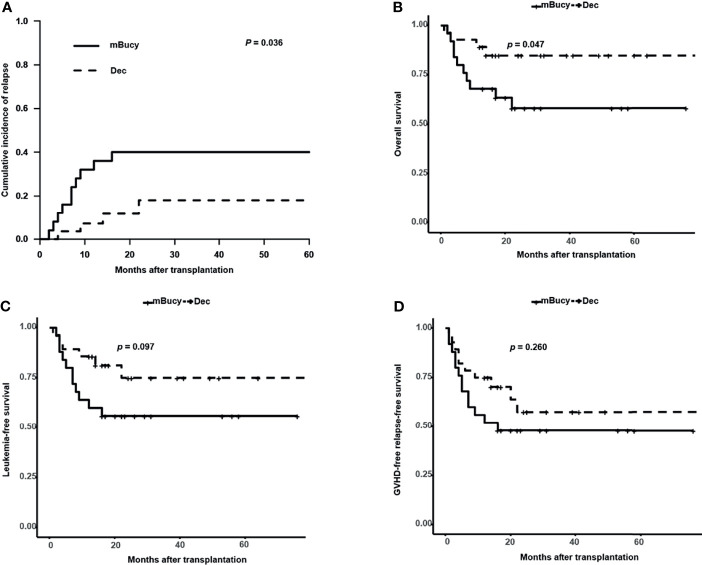
Outcomes in patients undergoing haploidentical donor (HID)-hematopoietic stem cell transplantation (HSCT). **(A)** Cumulative incidence of relapse. Probability of **(B)** overall survival, **(C)** leukemia-free survival, and **(D)** graft-versus-host disease (GVHD)-free relapse-free survival.

**Figure 5:**
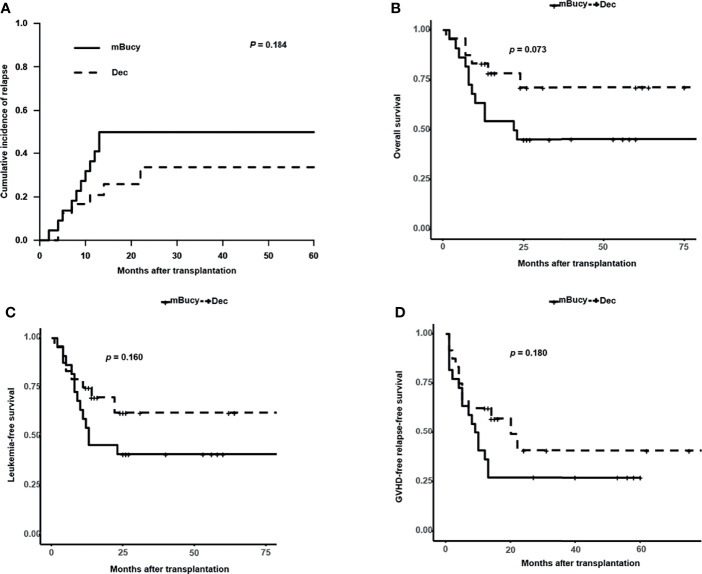
Outcomes in high-risk acute myeloid leukemia (AML) patients. **(A)** Cumulative incidence of relapse. Probability of **(B)** overall survival, **(C)** leukemia-free survival, and **(D)** graft-versus-host disease (GVHD)-free relapse-free survival.

Subgroup analysis of matched patients’ cohort was conducted according to the donor HLA type or risk stratification, and the number of patients in each subgroup was shown in **Table 1** (HID-HSCT subgroup: 28 vs. 25; ISD-HSCT: 18 vs. 21; high-risk subgroup: 24 vs. 22; intermediate-risk subgroup: 22 vs. 24). In the HID-HSCT subgroup, 2-year CIR was lower for the Dec group than for the mBuCy group (17.9% ± 0.8% vs. 40.0% ± 1.0%, *P* = 0.036; **Figure 4A**). There was a significant survival advantage for patients receiving Dec-containing conditioning, and 2-year OS of the Dec group was higher than that of mBuCy group (84.8% ± 7.1% vs. 58.2% ± 10.3%, *P* = 0.047; **Figure 4B**). Despite being not statistically significant (75.0% ± 9.3% vs. 56.0% ± 9.9%, *P* = 0.097; **Figure 4C**), Dec-intensified conditioning regimen exhibited an advantage in 2-year LFS, whereas 2-year GRFS was comparable between the two groups (57.5% ± 11.0% vs. 48% ± 10.0%, *P* = 0.260; **Figure 4D**). In the high-risk subgroup, Dec-intensified conditioning regimen tended to have a higher 2‐year OS (71.3% ± 10.4% vs. 45.5% ± 10.6%, *P* = 0.073; **Figure 5B**). No statistical differences were observed in 2-year CIR, LFS, and GRFS between the two groups (2-year CIR, 33.6% ± 1.3% vs. 50.0% ± 1.0%, *P* = 0.184; 2-year LFS: 62.2% ± 11.2% vs. 40.9% ± 10.5%, *P* = 0.160; 2-year GRFS: 40.9% ± 12.3% vs. 27.3% ± 9.5%, *P* = 0.180; **Figure 5**). Furthermore, in ISD-HSCT or intermediate-risk subgroup, 2-year CIR, OS, LFS, and GRFS were identical between the two groups (2-year CIR, 30.0% ± 1.4% vs. 29.7% ± 1.1%, *P* = 0.945, 11.0% ± 0.6% vs. 21.2% ± 0.8%, *P* = 0.279, respectively; 2-year OS, 75.8% ± 10.8% vs. 60.7% ± 10.9%, *P* = 0.340, 90.9% ± 6.1%, vs. 73.8% ± 9.3%, *P* = 0.170, respectively; 2-year LFS, 70.0% ± 11.5% vs. 55.9% ± 11.1%, *P* = 0.450, 84.4% ± 8.5% vs.70.4% ± 9.4%, *P* = 0.210, respectively; 2-year GRFS, 59.3% ± 12.1% vs. 31.7% ± 10.4%, *P* = 0.100, 75.5% ± 9.7% vs. 53.6% ± 10.3%, *P* = 0.089, respectively; **Figures S2, S3**).

## Discussion

To our knowledge, this retrospective matched‐pair study represents the first so far to evaluate the Dec-intensified mBuCy conditioning regimen in intermediate- and high-risk patients with newly diagnosed AML undergoing related donor HSCT.

The choice of Dec in combination with mBuCy was made mainly on the basis of 1) excellent antileukemic activity by inhibiting DNA methyltransferase (31), 2) strong synergistic cytotoxicity with Bu (32) or chemosensitization to Bu/Cy (33), and 3) favorable immunologic effects. The immune-modulating actions of Dec can increase the expression of silenced tumor-associated antigens and also of minor histocompatibility antigens for the donor immune system, contributing to graft-versus-leukemia (GVL) effect (34–36). Dec was found to decrease the incidence of GVHD (18, 37) that is likely due to increased numbers of regulatory T (Treg) cells (12, 38). Our propensity matched cohort study confirmed that adding Dec to the mBuCy regimen tended to reduce 2-year CIR and 2-year TRM. There was significantly decreased cumulative incidence of grade II–IV aGVHD in the Dec group than that in the mBuCy group. Therefore, patients who received Dec-intensified regimen were superior to those who received mBuCy in terms of 2-year OS and 2-year GRFS. Multivariate analyses also suggested that Dec was associated with longer OS, LFS, and GRFS. Our results compare favorably with previous reports of MAC regimens showing OS about 70% (39, 40). Compared to total body irradiation (TBI) or Bu-based fludarabine/amsacrine/cytarabine (FLAMSA) sequential intensified regimen (fludarabine, cytarabine, amsacrine, Cy, and either total body irradiation or Bu) in patients with intermediate- or high-risk AML in CR, the Dec-intensified regimen in this study displayed a higher 2-year OS and 2-year LFS (81.0% vs. 56.1%, 73.1% vs. 52.8%) (41). Our results also showed a survival benefit than an open-label phase II randomized trial (42) utilizing Bu-based FLAMSA regimen, associated with 2-year OS of 60.9% and 2-year LFS of 54.2%. Overall, Dec plus mBuCy regimen might provide better outcomes than the previous sequential intensified regimens.

Furthermore, the comparison provided evidence that Dec, as an attractive regimen in HID-HSCT setting, might yield synergistic GVL effects to achieve better survival without increasing the incidence of GVHD (data not shown). Remarkably, for high-risk AML patients, Dec-intensified regimen was superior to our previous study (22) using idarubicin-intensified HID-HSCT (3-year OS, 81.5% vs. 66.5%; 3-year LFS, 72.9% vs. 63.3%). Meanwhile, Dec-intensified HID-HSCT system resulted in an equivalent therapeutic effect as for ISD-HSCT patients (2-year OS, *P* = 0.570; 2-year LFS, *P* = 0.730; 2-year GRFS, *P* = 0.920), consistent with previous reports comparing outcomes of HID- to ISD-HSCT in AML patients in CR (43, 44). Although no difference in outcomes between two regimens was evident when analysis was restricted to patients undergoing ISD-HSCT, the cumulative incidence of grade II–IV aGVHD and extensive cGVHD was decreased (data not shown). Larger sample and longer follow-up are further needed to better interpret the impact of Dec-intensified conditioning regimen on ISD-HSCT outcomes. These results indicate that Dec-intensified conditioning might be a valid option for AML patients undergoing related donor transplantation but preferred for HID-HSCT.

In multivariate analysis, high-risk status was independently associated with all inferior outcomes. This study showed that low-dose Dec-containing conditioning did not overcome the bad prognosis of high-risk status but tended to have lower relapse rate and longer survival in high- or intermediate-risk patients compared with the mBuCy group; the small sample size and large differences in the proportion of patients with 2 or more high-risk characteristics between the two groups (Dec group, 45.8%; mBuCy group, 13.6%) may interfere with the conclusions. However, patients’ survival rate in this study was similar to that of previous studies. Tang et al. (10) retrospectively analyzed the use of Dec combined with mBuCy in high-risk AML patients and similarly reported higher 2-year OS rates and 2-year LFS rates compared to our study (78.3% vs. 71.3%, 66.8% vs. 62.2%). A multicenter prospective phase II study also showed that 1-year OS, LFS, and GRFS were 70%, 66%, and 45%, respectively, when adding 10-day Dec to fludarabine/2 Gy TBI (17). Compared to a recent study by Li et al. (45) (Dec 75 mg/m^2^ i.v. on day −9 and 50 mg/m^2^ i.v. on day −8), our treatment plan (Dec 20 mg/m^2^/day i.v. on days −11 to −7) in high-risk patients achieved higher 3-year OS and 3-year LFS (61.1% vs. 50.0%, 51.9% vs. 35.0%). This result, which is inconsistent with our study, may be attributable to the differences of Dec therapy and patient disease status pretransplantation. Additionally, in univariate analysis, the cumulative incidence of grade III–IV aGVHD and extensive cGVHD was not correlated with the outcomes possibly due to (1) small sample data (32.6% of patients with severe GVHD), which may have affected the statistical effect, and (2) timely prevention and treatment of GVHD in our center reduced the occurrence of adverse outcomes (**Table 3** showed that only 9.5% of patients died from GVHD).

More notably, previous research has shown that Dec had little hematological toxicities in allo-HSCT (10, 18, 19, 37). In our study, although the Dec group was associated with higher occurrence of oropharyngeal mucositis, it neither induced fatal/severe toxicity nor increased infectious episodes compared with mBuCy regimen. Only 10-day decitabine starting between 17 and 24 days prior to the conditioning regimen has been reported to be excessively toxic, increasing the association with infection in people at high risk of myeloid disease (46). These results provide rationale that the low-dose Dec (20 mg/m^2^/day for 5 days) potentiates the antileukemic effects while retaining sufficient tolerability.

However, some limitations of the present study should be considered. These include a single-center design, small sample size, and the relatively short follow-up time of some patients, which may reduce the reliability of the comparative inferences.

In conclusion, Dec-intensified mBuCy conditioning regimen may be a priority for intermediate- and high-risk AML patients, especially in patients undergoing HID-HSCT, as it is able to provide better survival and tolerated toxicity; certainly, a randomized clinical trial is required to provide more definition.

## Data Availability Statement

The original contributions presented in the study are included in the article/**Supplementary Material**. Further inquiries can be directed to the corresponding authors.

## Ethics Statement

The studies involving human participants were reviewed and approved by Institutional Review Board on Medical Ethics at Tongji Medical College of Huazhong University of Science and Technology. Written informed consent to participate in this study was provided by the participants’ legal guardian/next of kin.

## Author Contributions

YH and HFW designed the study. ZYL, WS, XL, HL, XNC, LT, HY, ZDZ, YY, and LHX selected and treated patients and contributed data. ZYL, WS, and XL analyzed the data and wrote the article. YH and HFW critically reviewed the article. All authors read and approved the final version of the article.

## Funding

This work was supported by the National Natural Science Foundation of China (No. 81770134, 82100233), Beijing Medical Award Foundation of China (YXJL-2019-0163-0114), and Chen Xiao-ping Foundation for the Development of Science and Technology of Hubei Province, China (CXPJJH12000009-114).

## Conflict of Interest

The authors declare that the research was conducted in the absence of any commercial or financial relationships that could be construed as a potential conflict of interest.

## Publisher’s Note

All claims expressed in this article are solely those of the authors and do not necessarily represent those of their affiliated organizations, or those of the publisher, the editors and the reviewers. Any product that may be evaluated in this article, or claim that may be made by its manufacturer, is not guaranteed or endorsed by the publisher.
