# Supplementary material for: Decitabine-Intensified Modified Busulfan/Cyclophosphamide Conditioning Regimen Improves Survival in Acute Myeloid Leukemia Patients Undergoing Related Donor Hematopoietic Stem Cell Transplantation: A Propensity Score Matched Analysis
Source: Front Oncol. 2022 Mar 16;12:844937. doi: 10.3389/fonc.2022.844937 (PMC8966032; doi:10.3389/fonc.2022.844937)
Supplement: Supplementary file 1 [file DataSheet_1.docx]

**Table S1** Characteristics of unmatched patients’ cohort.

| Variable | Dec (n=58) | mBuCy (n=98) | *P* value |
| --- | --- | --- | --- |
| Median age, years (range) | 34 (14-56) | 38 (13-61) | 0.540 |
| Sex (n, %) |  |  | 0.014 |
| Female | 13 (22.4) | 41 (41.8) |  |
| Male | 45 (77.6) | 57 (58.2) |  |
| Risk stratification (n, %) |  |  | ＜0.001 |
| Intermediate-risk | 22 (37.9) | 69 (70.4) |  |
| High-risk | 36 (62.1) | 29 (29.6) |  |
| Disease status before HSCT (n, %) |  |  | 0.555 |
| CR_MRD-_ | 51 (87.9) | 91 (92.8) |  |
| CR_MRD+_ | 4 (6.9) | 4 (4.2) |  |
| NR | 3 (5.2) | 3 (3.0) |  |
| Median number of prior lines of therapy (range) | 3 (1-8) | 3 (1-8) | 0.282 |
| Disease course before HSCT (n, %) |  |  | 0.294 |
| ≤12months | 53 (91.4) | 94 (95.9) |  |
| >12months | 5 (8.6) | 4 (4.1) |  |
| Donor age, years, median (range) | 41 (9-58) | 34 (10-57) | 0.181 |
| Donor/recipient sex match (n, %) |  |  | 0.382 |
| Female-male | 16 (27.6) | 21 (21.4) |  |
| No female-male | 42 (72.4) | 77 (78.6) |  |
| ABO match (n, %) |  |  | 0.776 |
| Mathed | 33 (56.9) | 61 (62.3) |  |
| Major mismatched | 12 (20.7) | 21 (21.4) |  |
| Minor mismatched | 9 (15.5) | 12 (12.2) |  |
| Bidirect mismatched | 4 (6.9) | 4 (4.1) |  |
| Donor HLA type (n, %) |  |  | 0.028 |
| HLA-identical sibling | 18 (31.0) | 48 (49.0) |  |
| HLA-antigen mismatched related | 40 (69.0) | 50 (51.0) |  |
| Stem cell source (n, %) |  |  | 0.222 |
| PBSC | 46 (79.3) | 85 (86.7) |  |
| PBSC+BM | 12 (20.7) | 13 (13.3) |  |
| Median follow-up time, months, (range) | 21 (1-85) | 25 (1-109) | 0.218 |

Dec, decitabine; mBuCy, modified busulfan/cyclophosphamide; HSCT, hematopoietic stem cell transplantation; CR, complete remission; MRD, minimal residual disease; NR, active disease; PBSC, peripheral blood stem cell; BM, bone marrow.

**
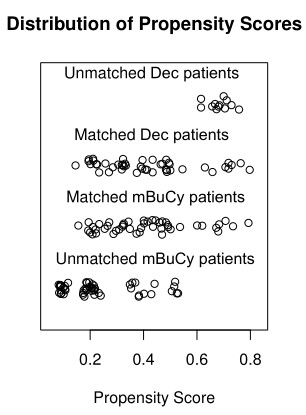
**

**Figure S1** Distributions of propensity scores for the unmatched and matched patients’ cohorts

**
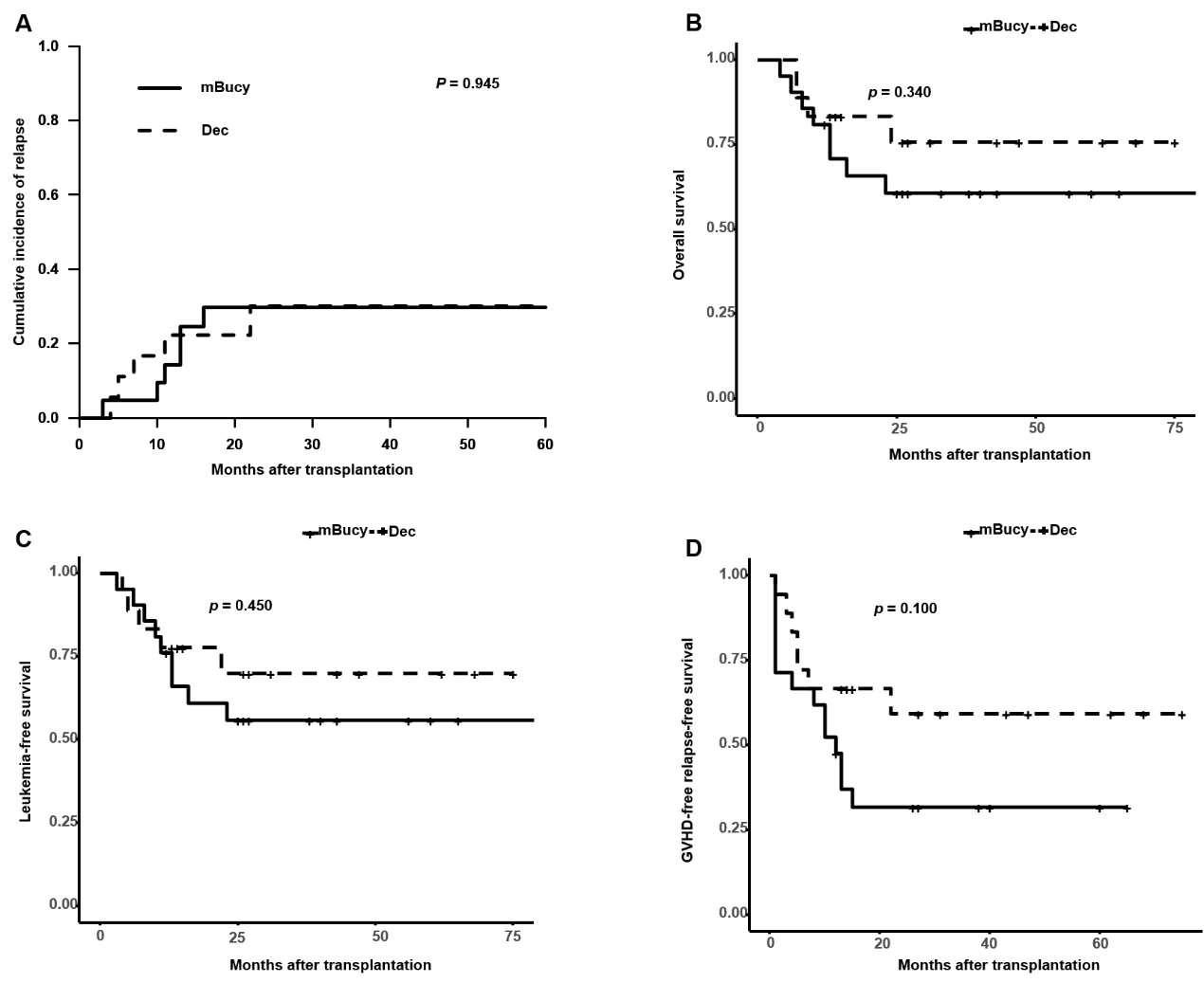
**

**Figure S2** Outcomes in patients undergoing ISD-HSCT. (A) Cumulative incidence of relapse. Probability of (B) overall survival, (C) leukemia-free survival, and (D) GVHD-free relapse-free survival.

**
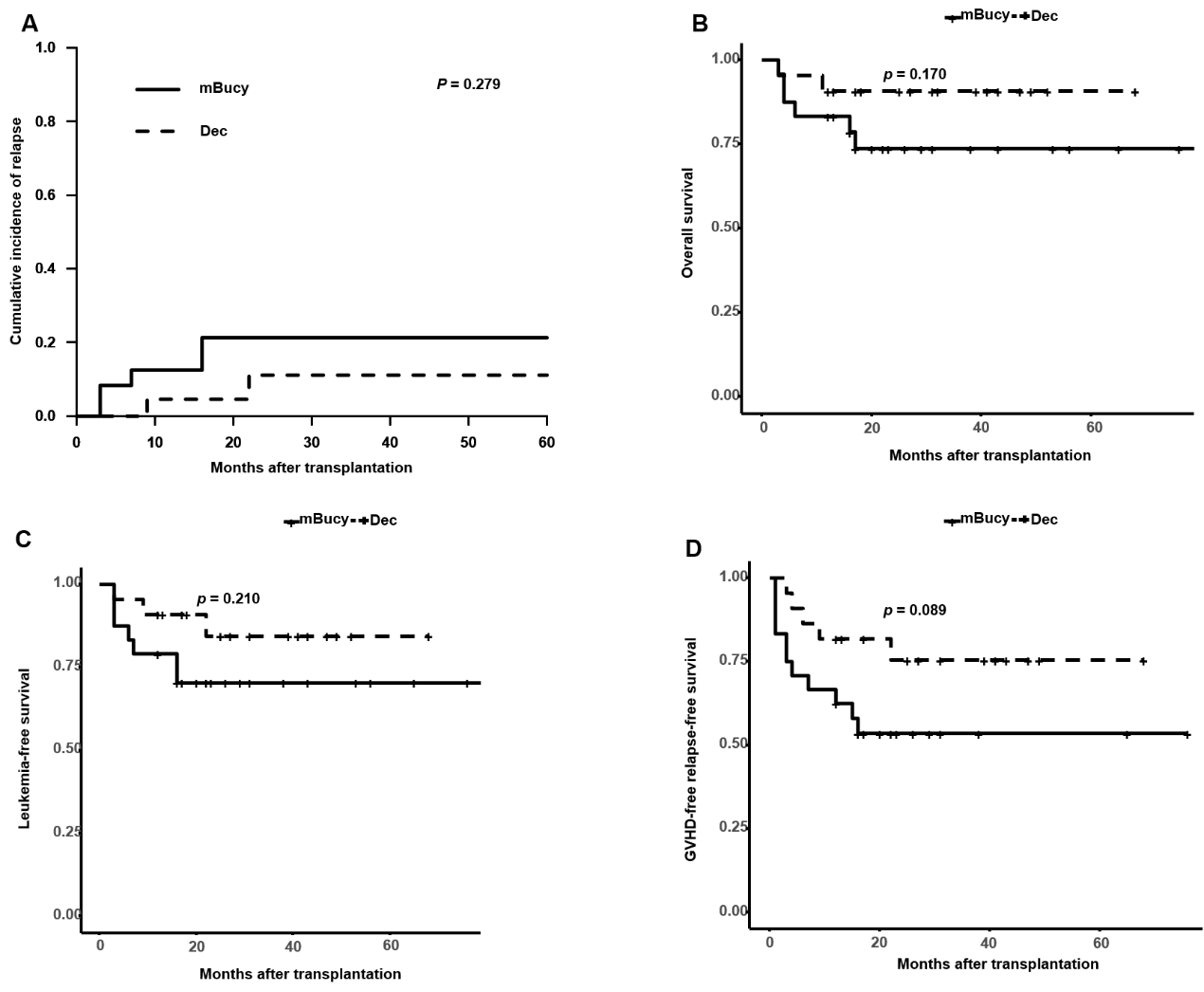
**

**Figure S3.** Outcomes in intermediate-risk AML patients. (A) Cumulative incidence of relapse. Probability of (B) overall survival, (C) leukemia-free survival, and (D) GVHD-free relapse-free survival.
